# Unraveling the hydraulic vulnerability of tree seedlings using optical and acoustic techniques

**DOI:** 10.1093/jxb/erag039

**Published:** 2026-01-29

**Authors:** Adriano Losso, Andrea Ganthaler, Stefan Mayr, Barbara Beikircher

**Affiliations:** Department of Botany, University of Innsbruck, Innsbruck A-6020, Austria; Department of Botany, University of Innsbruck, Innsbruck A-6020, Austria; Department of Botany, University of Innsbruck, Innsbruck A-6020, Austria; Department of Botany, University of Innsbruck, Innsbruck A-6020, Austria; Ghent University, Belgium

**Keywords:** Acoustic emissions, hypocotyl, optical technique, tree seedlings, vulnerability to drought, xylem embolism

## Abstract

The seedling stage is critical for tree recruitment and forest regeneration, but faces high mortality rates, with drought being a major cause. However, knowledge of the hydraulic vulnerability of tree seedlings is scarce due to methodological difficulties related to their small size. We quantified the xylem vulnerability of the hypocotyl to drought-induced embolism using the ultrasonic acoustic emission and optical visualization techniques by performing simultaneous measurements on dehydrating 5- to 8-week-old seedlings of *Acer pseudoplatanus*, *Sorbus aucuparia*, *Larix decidua*, and *Pinus cembra*. Optical visualization was also used on the angiosperm leaves. Species-specific differences in hypocotyl and leaf vulnerability were observed. Acoustic emission data showed that the hypocotyl vulnerability of *S. aucuparia*, *L. decidua*, and *P. cembra* was similar to that reported for mature tree branches. Optical visualization was similar to acoustic emission vulnerability in *A. pseudoplatanus* and *P. cembra*, but higher in *L. decidua* and *S. aucuparia* (with differences of 0.83 and 2.50 MPa, respectively). The latter showed exceptional higher frequencies in small conduits, which may be difficult to observe with optical visualization. Both techniques can be used to provide new insights into tree seedling hydraulics, which will be crucial for better predicting forest regeneration in the face of climate change.

## Teaser

The hypocotyls of tree seedlings showed a vulnerability to drought-induced xylem embolism that was surprisingly similar to that of branches of mature trees in three out of four species.

## Introduction

A tree can live for hundreds of years, during which time it must be able to withstand and adjust to the changing and sometimes harsh environmental conditions occurring at the site of its germination. In particular, the first few weeks after germination are characterized by significant morphological and related physiological changes (e.g. Miller and Johnson, 2017; Miller *et al*., 2020; Beikircher *et al*., 2025). For a newly established tree seedling, the seed is still the main source of energy until the cotyledons unfold and the plant can start photosynthesizing. However, even after the seedling has become self-sustainable and independent of the seed, in the first stages of development it remain at high risk of death due to low productivity, limited reserves (Fenner, 1987; Smith *et al*., 2003; Johnson *et al*., 2011), and water supply. During the first few months of development, water relations are often key to the survival of seedlings, as they have limited hydraulic capacitance and small root systems, making them dependent on water from the upper layers of the soil, which can easily become restricted due to dehydration. Accordingly, the recent increase in intensity and frequency of late spring (e.g. Brázdil *et al*., 2015; Spinoni *et al*., 2017) and summer droughts (e.g. Schuldt *et al*., 2020; Arend *et al*., 2021) will have a critical effect on tree seedling recruitment and survival (e.g. Facelli, 2008; Johnson *et al*., 2011; Augustine and Reinhardt, 2019; Losso *et al*., 2019). This is all the more important as seedlings play a major role in forest population dynamics and shifts in species distributions under climate change (Ibanez *et al*., 2007; Vanderwel *et al*., 2013), making knowledge of seedling hydraulics relevant to a variety of fields, including forestry and nature conservation.

In plants, transpiring leaves create negative pressures that are transmitted down to the roots, allowing water uptake and root-to-leaf transport within the xylem network. This water is in a metastable state (see the cohesion–tension theory; Tyree and Zimmermann, 2002) and therefore at risk of transition from liquid to vapor (i.e. xylem embolism; Cochard, 2006). Xylem embolism occurs when tension in the sap causes gas bubbles to be aspirated through the pits into adjacent xylem conduits (air seeding; Tyree and Zimmermann, 2002), and the main cause in nature is drought stress. Vulnerability to xylem embolism is thus critical to plant survival, as embolisms can spread throughout the xylem network, reducing hydraulic conductance and consequently plant productivity and, in extreme cases, leading to plant death (Sperry *et al*., 1993; Brodribb and Cochard, 2009). Notably, the hydraulic vulnerability to drought-induced xylem embolism of seedlings has been little studied, mainly because of the methodical difficulties posed by their small size. To our knowledge only a few studies have actually tested it in seedlings younger than 6 months (Losso *et al*., 2019; Beikircher *et al*., 2025).

The vulnerability of plants to drought-induced xylem embolism can be quantified using vulnerability curves, which describe the progressive impairment of the xylem hydraulic network as a function of the water potential (Ψ) (e.g. Sperry *et al*., 1988). Within the plant hydraulic research community, several techniques have been developed to construct vulnerability curves for different plant species as well as plant organs (e.g. Sperry *et al*., 1988; Brodribb and Holbrook, 2006; Hietz *et al*., 2008; Brodersen *et al*., 2010; Nolf *et al*., 2015; Losso *et al*., 2016, 2019; Brodribb *et al*., 2017; Rodriguez-Dominguez *et al*., 2018; Feng *et al*., 2021), but most of them require large plant material and are not suitable for small material such as seedlings a few weeks old (Beikircher *et al*., 2021, 2025). Two techniques that have already been used to analyse vulnerability to drought-induced xylem embolism in small plants are ultrasonic acoustic emission (AE) analysis and optical visualization (OV). AE can record acoustic signals emitted during air seeding as samples dry (Tyree and Sperry, 1989; Ponomarenko *et al*., 2014; Nolf *et al*., 2015), while OV can record xylem embolism-induced changes under light transmission in dehydrating samples (Brodribb *et al*., 2016, 2017). AE is a technique that has been used for decades (e.g. Nardini *et al*., 2024), and has shown good agreement with other standard hydraulic techniques (especially in conifers; e.g. Tyree and Dixon, 1983; Cochard, 1992; Hacke and Sauter, 1995; Mayr and Rosner, 2011; Losso *et al*., 2025). OV has been more recently developed, and has also shown good agreement with other standard techniques as long as small samples are analysed (<0.15–0.3 mm; Brodribb *et al*., 2017; Johnson *et al*., 2020; Losso *et al*., 2025).

In this study, we analysed seedlings of four alpine angiosperm (*Acer pseudoplatanus* L. and *Sorbus aucuparia* L.) and conifer (*Larix decidua* Mill. and *Pinus cembra* L.) trees with regard to the vulnerability to drought-induced xylem embolism of their hypocotyls with both AE and OV. We also tested the hydraulic vulnerability of the leaves of the two angiosperms with OV. In addition, we measured hydraulically important anatomical traits, such as xylem width, mean (*d*) and hydraulic (*d*_h_) diameter of conduits and conduit wall reinforcement [i.e. conduit wall thickness to span ratio (*t*/*b*)^2^] in the hypocotyl. We hypothesized that AE and OV measurements would show similar vulnerability and that seedlings would be less hydraulically safe than their mature counterparts (when compared with previous studies). We also expected to observe general differences between angiosperms and conifers, particularly due to their xylem anatomy, as well as differences between the hydraulic vulnerability of angiosperm hypocotyls and leaves.

## Material and methods

### Plant material

All experiments were conducted between June and July 2023 on 5- to 8-week-old seedlings of two angiosperms (*Acer pseudoplatanus* L. and *Sorbus aucuparia* L.) and a deciduous (*Larix decidua* Mill.) and an evergreen (*Pinus cembra* L.) conifer. In May 2023, cold stratified seeds of all species were sown in small pots (0.27 liters, 7×7×8 cm), filled with a mixture of soil designed for alpine plants (leaf mold: ground soil: coconut fiber: sand: horticultural lava: rock meal, 5: 2: 2: 2: 1: 0.15). To ensure optimal growth conditions, the plants were placed in a greenhouse in the Innsbruck Botanical Garden, Austria, and watered daily. At the time of measurements, seedlings were about 3.7–6.7 cm tall with a basal hypocotyl diameter of 0.8–1.7 mm (Supplementary Table S1), with cotyledons and two to four true leaves (angiosperms), or 10–25 needles (conifers).

### Vulnerability analyses

#### Acoustic emissions analyses

For the AE technique (Wolkerstorfer *et al*., 2012; Cochard *et al*., 2013; Lamacque *et al*., 2022), fully hydrated seedlings (*n*=6–8 per species) were removed from the pots, soil residues were carefully removed from roots, and hypocotyls were prepared by removing a short section of the periderm (ca. 2 cm long and as wide as the hypocotyl diameter). The exposed xylem was covered with silicone grease to prevent any further water loss and optimize acoustic coupling, and a 150 kHz resonance acoustic sensor (R15) connected to a preamplifier set to 40 dB (Physical Acoustics, Wolfegg, Germany) was tightly clamped to the hypocotyl of each seedling. AE was recorded using an eight-channel system (PAC Micro-II Express Digital AE System, Physical Acoustics) with a threshold of 35 dB and AEwin software (Mistras Holdings Corp., Princeton, NJ, USA). Another set of seedlings (ca. 50–60 per species) were placed in between those with acoustic sensors (Supplementary Fig. S1A) and used to measure the plant water potential (Ψ). To avoid excessive transpiration and to maintain a balanced Ψ within plants, seedlings were covered with black plastic bags and then bench-dehydrated for up to 20 h (i.e. when Ψ was no longer measurable). At regular time intervals, two to three plants per species were placed in small plastic bags and, after a 5–10 min equilibration period, cut at the root collar and Ψ measured using a Scholander apparatus (model 1505D pressure chamber; PMS Instrument Co., Albany, OR, USA). Preliminary tests have shown that dehydration of seedlings of the same size under black plastic bags is uniform. To obtain the Ψ at any given time, linear interpolations between each two consecutive measured potentials were computed (see Nolf *et al*., 2015).

As AE can also detect embolism formation in fibers, emissions from non-conducting cells and tissues, or emissions from wood microfractures (see Nolf *et al*., 2015; Rosner, 2015; Lamacque *et al*., 2022), acoustic emissions often do not plateau after complete dehydration, making it difficult to construct vulnerability curves (see Beikircher *et al*., 2025). Instead, acoustic activity (signals min^−1^) was calculated for each individual in 1 min increments and plotted *versus* the corresponding Ψ. We then determined the Ψ at maximum acoustic activity (Ψ_AEmax_), which reflects the Ψ at maximum embolism formation and thus to 50% of loss of hydraulic conductivity (Ψ_50_), by fitting the Savitzky–Golay smoothing function (Savitzky and Golay, 1964) to emission rates (see Nolf *et al*., 2015; Beikircher *et al*., 2025) to obtain stable and comparable results. Individual values were then averaged per species (mean ±standard error).

#### Optical visualization

In parallel with the AE analyses, we also analysed hypocotyl and leaf (only for the angiosperms *A. pseudoplatanus* and *S. aucuparia*) vulnerability using the OV technique (Brodribb *et al*., 2016, 2017). As for AE analysis, fully hydrated seedlings (*n*=5–8 per species) were removed from pots, soil residues were carefully removed, and a short hypocotyl section (ca. 2 cm long and as wide as the hypocotyl diameter) was decorticated. The exposed xylem was covered with a conductive adhesive gel (Aquasonic Clear; Parker Laboratories Inc., Fairfield, NJ, USA) to reduce heterogeneity in the rate of desiccation between different xylem layers and to improve light transmission. Custom-made clamps (http://www.opensourceov.org) containing an 8 megapixel Raspberry Pi Camera v2 connected to a Raspberry Pi single board computer (Raspberry Pi Foundation, Cambridge, UK) and light emitting diodes (LEDs) were placed with the camera facing the exposed xylem. This setup allowed images to be acquired with sufficient spatial resolution to detect changes in light reflectance (for hypocotyls) or transmittance (for leaves) during embolism formation (e.g. Brodribb *et al*., 2016). The Raspberry Pi single board computer was set to capture images every 150 s. For angiosperms, an additional clamp was attached to the first true leaves of *A. pseudoplatanus* and to the terminal leaflets of *S. aucuparia* compound leaves (Supplementary Fig. S1), and the same settings were used. The terminal leaflet of *S. aucuparia* was chosen for two reasons: its larger size and the possibility of placing two clamps on the same small seedling. As for AE, another set of seedlings (ca. 50–60 per species) was placed in between those with clamps (Supplementary Fig. S1B) and used to measure Ψ. All samples were then placed under plastic bags and dehydrated for up to 20 h (i.e. when Ψ was no longer measurable). Ψ at any given time was obtained as described above.

Images were downloaded and analysed using Fiji (a Java-based version of ImageJ; Schindelin *et al*., 2012) following established protocols (https://github.com/OpenSourceOV) that subtract successive images showing contrast changes caused by embolism formation. The percentage of embolized hypocotyl/leaf xylem area was determined as:


(1) $$\text{\%}\;\mathrm{of}\;\mathrm{embolized}\;\mathrm{xylem}\;\mathrm{area}=\frac{A_{\mathrm{cum}}}{A_{\max}}\times 100$$

where *A*_cum_ is the cumulative area of embolized xylem at any given time and *A*_max_ is the maximum area of embolized xylem at the end of dehydration. Ψ was coupled with the corresponding cumulative embolized area, and vulnerability curves were then fitted to a Weibull function using the R package fitplc (Duursma and Choat, 2017). Water potentials corresponding to 12%, 50%, and 88% of embolized pixel area (Ψ_OV12_, Ψ_OV50_, and Ψ_OV88_, respectively) and their 95% confidence intervals were extracted from the vulnerability curves using a standard profiling method.

### Anatomy

Hypocotyls from the same seedling cohort used for vulnerability analysis (at least five per species) were embedded in paraffin and cut at 10 µm using a rotary microtome (CUT 5062, Slee medical GmbH, Nieder-Olm, Germany) in the region where OV and AE analyses were performed. Cross and longitudinal sections were stained with safranin and astra blue and permanently fixed using Euparal following standard protocols (Von Arx *et al*., 2016). Images were captured using a light microscope (Olympus BX 41 system microscope, Austria) connected to a digital microscope camera (ProgRes CT3, Jenoptik, Jena, Germany). Cross-section images were then analysed using ImageJ 1.54 software (National Institutes of Health, Bethesda, MD, USA).

The width of the xylem was measured at each cardinal point on five samples per species, and averages were then used to calculate means and SE per species. For each sample, conduit lumen areas were measured and diameters (*d*; μm) were calculated assuming square shapes for conifers and circular shapes for angiosperms (Tomasella *et al*., 2018). To avoid unbalanced statistical weighting of samples with larger numbers of analysed conduits, diameters were first averaged for each hypocotyl sample and then for each species to obtain mean *d* (*d*_mean_; Beikircher *et al*., 2021). Based on Sperry *et al*. (1994), mean hydraulic diameter (*d*_h_) was then calculated as:


(2) $$d_{h}=\;\sum d^{5}/\;\sum d^{4}$$

For each hypocotyl section, the cell wall reinforcement [(*t*/*b*)^2^] (Hacke *et al*., 2001) was estimated by measuring the double wall thickness (*t*) and the conduit diameter (*b*) in at least 20 conduit pairs with diameters within *d*_h_±1 μm (Hacke and Sperry, 2001; Hacke *et al*., 2001). As for *d*_mean_, *d*_h_ and (*t*/*b*)^2^ were also calculated first for each sample and then averaged per species.

### Statistics

For comparisons between techniques (i.e. Ψ_AEmax_ versus Ψ_OV50_), between Ψ_AEmax_ of different species, and anatomical parameters, differences were tested using one-way ANOVA followed by Tukey’s post-hoc test after testing for normal distribution and variance (Shapiro–Levene test). For OV vulnerability analyses, differences between organs were assessed using 95% confidence intervals obtained by bootstrap resampling. We also assessed the relationships between anatomical traits [xylem thickness and (*t*/*b*)^2^] and vulnerability thresholds (Ψ_AEmax_ and Ψ_OV50_) by generating a correlation plot using Pearson’s correlation coefficient. All statistical data were analysed using R 4.4.1 (R Core Team, 2017) at the 5% probability level.

## Results

### Vulnerability analyses

**Fig. 1.**
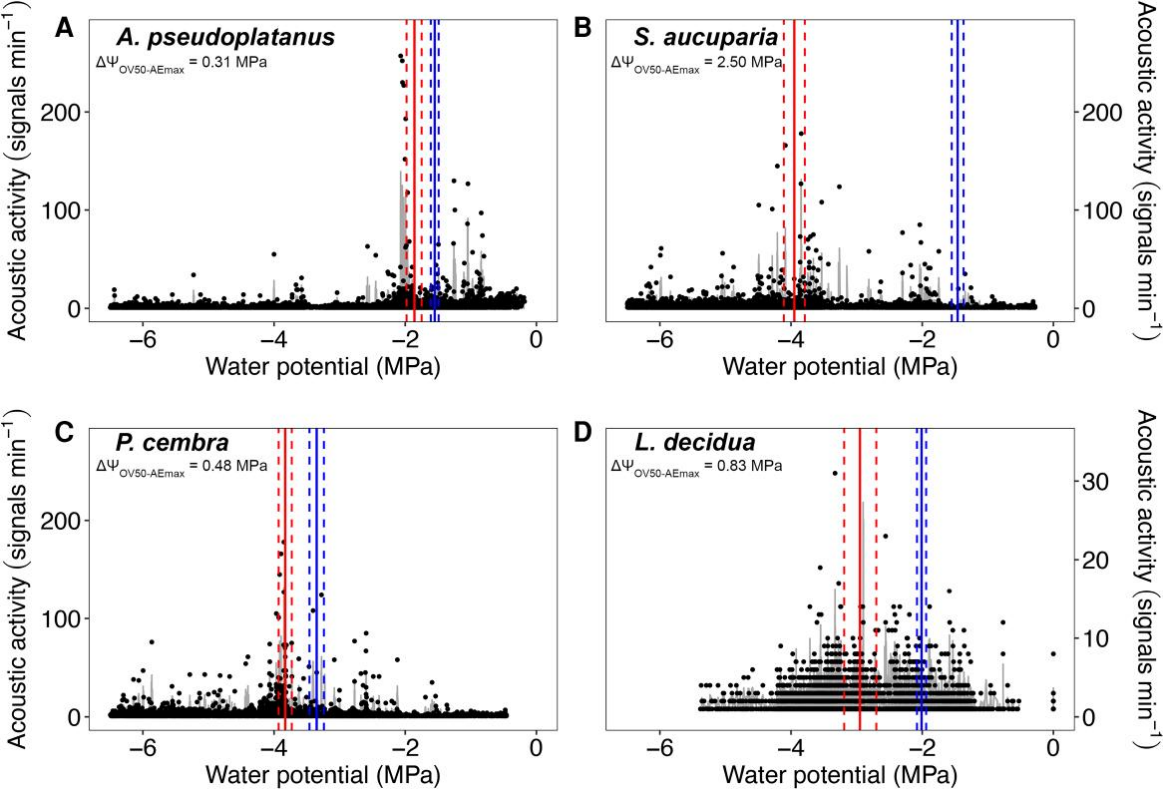
Acoustic activity versus plant water potential of dehydrating seedlings. Acoustic activity (signals min^−1^) versus plant water potential (Ψ, MPa) of seedlings of *Acer pseudoplatanus* (A; *n*=8), *Sorbus aucuparia* (B; *n*=8), *Pinus cembra* (C; *n*=8), and *Larix decidua* (D; *n*=6). Red vertical lines represent the Ψ at the main peak of acoustic activity (Ψ_AEmax_). For comparison, blue vertical lines represent the Ψ at 50% of embolized pixel area (Ψ_OV50_; see Fig. 2). Dashed lines indicate the standard errors (SE) of Ψ_AEmax_ and Ψ_OV50_. Grey lines indicate ﻿Savitzky–Golay smoothed values. The difference between the two parameters (ΔΨ_OV50−AEmax_) is also given. Note different scale for the *y*-axis in *L. decidua* (D).

Acoustic analyses revealed significant inter-specific differences in hypocotyl vulnerability. The Ψ at maximum acoustic activity (Ψ_AEmax_; Fig. 1; Supplementary Table S2) indicated *S. aucuparia* (Ψ_AEmax_ −3.95±0.16 MPa) and *P. cembra* (Ψ_AEmax_ −3.83±0.10 MPa) to be the most resistant species, whereas *A. pseudoplatanus* was the least resistant (Ψ_AEmax_ −2.01±0.07 MPa).

**Fig. 2.**
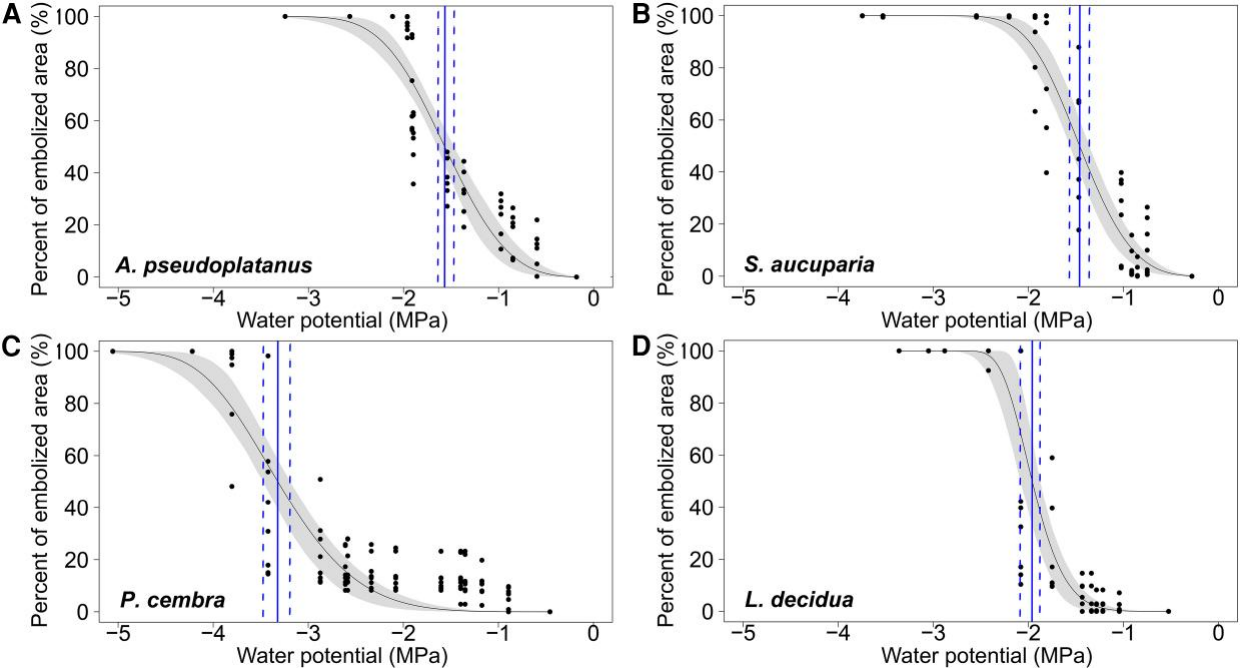
Percentage of embolized xylem area versus plant water potential of dehydrating seedlings. Percentage of embolized xylem area (%) versus plant water potential (Ψ, MPa) of seedlings of *Acer pseudoplatanus* (A; *n*=6), *Sorbus aucuparia* (B; *n*=7), *Pinus cembra* (C; *n*=8), and *Larix decidua* (D; *n*=7) measured with OV. Blue vertical lines represent the Ψ_OV50_ (solid line) and the 95% confidence interval (CI, dashed lines). Grey area represents the CI of the curve.

OV vulnerability curves also showed that hypocotyl vulnerability differs between species (Fig. 2; Supplementary Fig. S2; Table 1), but Ψ_OV50_ was overall less negative in the two angiosperms (*A. pseudoplatanus* −1.56 MPa, *S. aucuparia* −1.46 MPa) than in the two conifers (*L. decidua* −1.96 MPa), with *P. cembra* showing the lowest Ψ_OV50_ (−3.32 MPa). Species-specific differences in Ψ_OV12_ and Ψ_OV88_ mirrored those of Ψ_OV50_ (see Table 1). For all species under study, Ψ_OV50_ was always less negative than Ψ_AEmax_, but the difference (ΔΨ_OV50−AEmax_) was only significant for *S. aucuparia* (ΔΨ_OV50−AEmax_ 2.50 MPa; *P*<0.001) and *L. decidua* (ΔΨ_OV50−AEmax_ 0.83 MPa; *P*=0.001) (Fig. 1; Supplementary Table S2).

**Table 1.** Optical visualization vulnerability thresholds (i.e. Ψ_OV12_, Ψ_OV50_, and Ψ_OV88_) and 95% confidence intervals (CI) of hypocotyls and leaves of *Acer pseudoplatanus*, *Sorbus aucuparia*, *Pinus cembra*, and *Larix decidua* seedlings

Organ	Ψ_OV12_ (CI 2.5%, 97.5%) (MPa)	Ψ_OV50_ (CI 2.5%, 97.5%) (MPa)	Ψ_OV88_ (CI 2.5%, 97.5%) (MPa)	*n*
*A. pseudoplatanus*				
Hypocotyl	−0.96 (−0.81, −1.14) a*	−1.56 (−1.48, −1.65) a*	−2.16 (−2.02, −2.32) ab	6
Leaf	−1.90 (−1.87, −1.92) A	−1.95 (−1.94, −2.00) A	−1.98 (−1.97, −2.09) A	5
*S. aucuparia*				
Hypocotyl	−0.94 (−0.84, −1.09) a*	−1.46 (1.36, −1.56) a*	−1.95 (1.79, −2.07) b*	7
Leaf	−0.68 (−0.46, −0.79) B	−0.81 (0.67, 0.85) B	−0.90 (−0.86, −0.94) B	7
*P. cembra*				
Hypocotyl	−2.49 (−2.28, −2.77) b	−3.32 (−3.19, −3.47) b	−4.02 (−3.79, −4.24) c	8
*L. decidua*				
Hypocotyl	−1.60 (−1.50, −1.75) c	−1.96 (−1.87, −2.07) c	−2.24 (−2.08, −2.38) a	7

Values are the mean ±SE and *n* indicates the number of replicates. Different lowercase and capital letters indicate statistically significant differences between species hypocotyls and leaves, respectively. Asterisks indicate significant differences between hypocotyls and leaves of the same species.

**Fig. 3.**
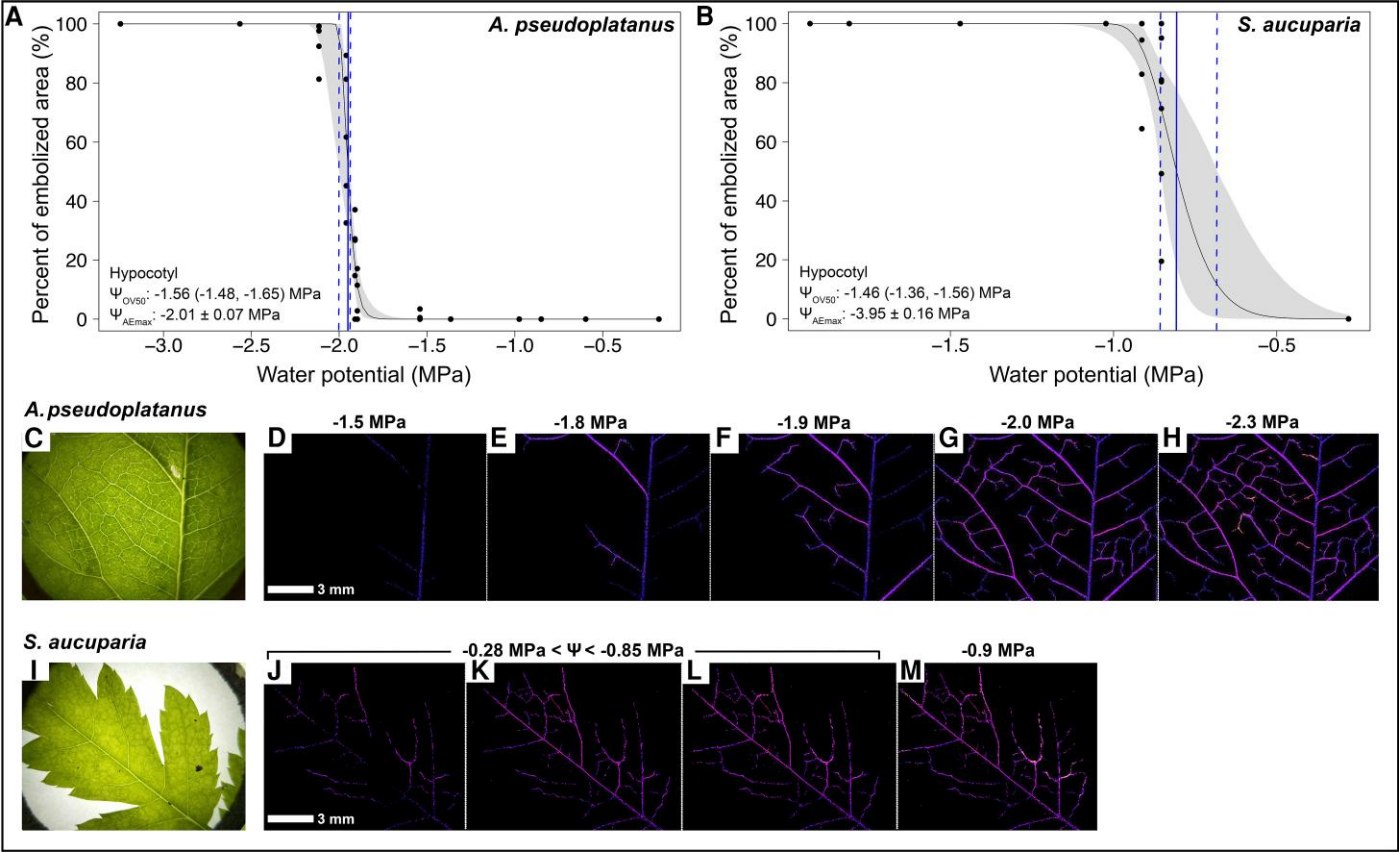
Percentage of embolized xylem area versus plant water potential of dehydrating leaves of seedlings. (A, B) Percentage of embolized xylem area (%) versus water potential (Ψ, MPa) of leaves of seedlings of *Acer pseudoplatanus* (A; *n*=5) and *Sorbus aucuparia* (B; *n*=7) obtained with the OV technique. Blue vertical lines represent the Ψ_OV50_ (solid lines) and the 95% confidence interval (CI, dashed lines). Grey area represents the CI of the curve. For comparison, both hypocotyl Ψ_OV50_ (with 95% CI) and Ψ_AEmax_ (±SE) are also given for each species (A, B). Note different scales for *x*- and *y*-axes. (C–M) Normal photos (C, I) and visual representation of the embolized xylem of the first true leaves (D–H) of *A. pseudoplatanus* and the terminal leaflets of *S. aucuparia* (J–M) ﻿recorded during dehydration by OV at different Ψ (MPa). For *S. aucuparia*, the Ψ is given as a range in (J–L) due to the rapid dehydration time (see also panel B). White bars in (D, I) indicate a 3 mm reference scale.

Leaf vulnerability analysis revealed that *A. pseudoplatanus* had a lower Ψ_OV50_ (−1.95 MPa) than *S. aucuparia* (−0.81 MPa) (Fig. 3; Table 1). While leaf Ψ_OV50_ was 0.4 MPa lower than hypocotyl Ψ_OV50_ in *A. pseudoplatanus*, it was 0.7 MPa less negative in *S. aucuparia* (Table 1).

### Anatomy

The seedlings of all species analysed showed a closed ring of xylem consisting of several rows of conduits. In *P. cembra*, resin channels surrounded by parenchyma interrupted the xylem ring (Supplementary Fig. S3). The width of the xylem was similar for all species, ranging from 85 µm to 96 µm (Table 2), but *A. pseudoplatanus* and *L. decidua* had the largest and smallest xylem area, respectively (Table 2).

**Table 2.** Xylem ring width, xylem area, xylem area proportion compared with other tissues (bark, pith), number of xylem conduits per cross-section, mean conduit diameter (*d*_mean_), mean conduit hydraulic diameter (*d*_h_), and cell wall reinforcement [(*t*/*b*)^2^] of hypocotyls of *Acer pseudoplatanus*, *Sorbus aucuparia*, *Pinus cembra*, and *Larix decidua* seedlings

	Xylem width (µm)	Xylem area (µm^2^)	Xylem area proportion (%)	Conduits (*n*)	*d*_mean_ (µm)	*d_h_*, µm	(*t*/*b*)²
*A. pseudoplatanus*	96.16±12.72 a	124 106.4±21 817.9 a	7.88±0.92 a	1821.0±383.7 a	7.01±0.75 a	17.91±3.97 a	0.052±0.003 a
*S. aucuparia*	85.07±12.21 a	76 952.0±10 659.4 ab	9.79±1.73 ab	2168.0±231.3 a	6.83±1.51 a	18.58±3.76 a	0.088±0.010 a
*P. cembra*	90.41±10.44 a	76 970.1±11 716.3 ab	4.97±0.53 a	732.6±116.2 b	6.87±0.36 a	9.00±0.65 b	0.123±0.007 b
*L. decidua*	96.23±4.38 a	36 101.8±2114.5 b	14.50±1.27 b	634.4±39.4 b	5.61±0.40 a	7.58±0.32 b	0.072±0.006 a

Values are the mean ±SE. Different letters indicate statistically significant differences between species (*n*=5).

**Fig. 4.**
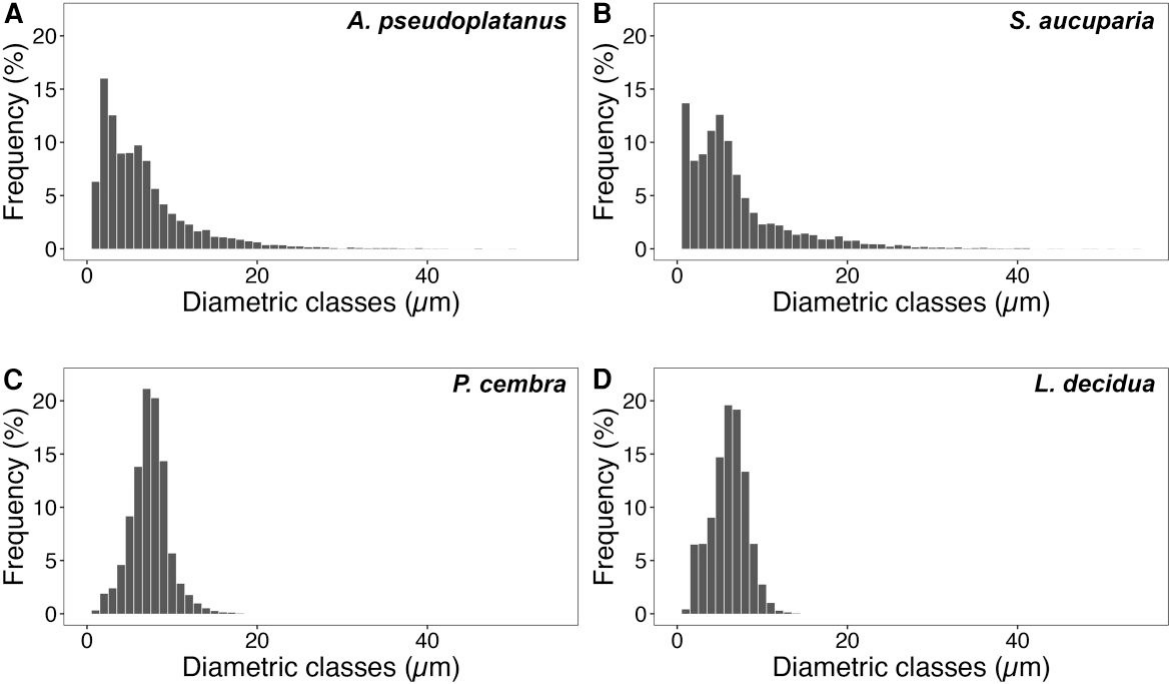
Distribution of conduit diameters (1 µm classes) of seedling hypocotyls of *Acer pseudoplatanus* (A), *Sorbus aucuparia* (B), *Pinus cembra* (C), and *Larix decidua* (D).

Mean conduit diameter (*d*_mean_) was highest in *A. pseudoplatanus* (7.01±0.75 µm) and lowest in *L. decidua* (5.61±0.40 µm), and the two angiosperm species had a significantly higher *d*_h_ than the two conifers (ca. 2-fold higher) (Table 2; Supplementary Fig. S3). The frequency distribution of conduit diametric classes showed angiosperms to have a higher frequency of large diameter conduits (15–45 μm) and fewer conduits between 6 μm and 9 μm than conifers (Fig. 4). *Sorbus aucuparia* also had a relatively high frequency of small diameter conduits (1 μm; ca. 14%) compared with other diametric classes and the other species (Fig. 4). Overall, the angiosperms also had a significantly higher number of conduits per cross-section (ca. 1800–2100) when compared with the conifers (ca. 630–730; Table 2). The xylem area of *L. decidua* covered a larger proportion of the cross-section (ca. 15%) than the other three species (<10%) (see also Supplementary Fig. S3).

Cell wall reinforcement [(*t*/*b*)^2^] was up to 2.3-fold higher in *P. cembra* (0.123±0.007) when compared with the other three species (Table 2). Xylem thickness and (*t*/*b*)^2^ showed no correlation either with ΔΨ_OV50−AEmax_ or with Ψ_OV50_ and Ψ_AEmax_.

## Discussion

This is the first study to show that both the optical visualization (OV) and the acoustic emission (AE) techniques can be used to analyse the hydraulic vulnerability to drought-induced xylem embolism of small and fragile tree seedlings. However, we also found an inconsistency between the two techniques, with OV reporting an overall higher hypocotyl vulnerability than AE (particularly relevant for *S. aucuparia*), which could be related to the anatomical characteristics of these small plants (see following paragraph). Regardless of the technique used, vulnerability data for both hypocotyls and leaves revealed significant differences between study species (up to 1.9 MPa). Contrary to our hypotheses, the hypocotyl vulnerability measured using the acoustic technique was remarkably similar to that reported for the branches of mature trees in three of the four species. In *A. pseudoplatanus*, leaf veins were less vulnerable than hypocotyls, whereas the opposite was observed in *S. aucuparia*.

### Technique comparison

In recent years, most techniques for testing the vulnerability to drought-induced embolism of tree species, including AE and OV, have been compared with each other and with more established techniques such as the flow centrifuge technique and X-ray microtomography (Brodribb *et al*., 2017; Venturas *et al*., 2019; Gauthey *et al*., 2020; Pratt *et al*., 2020; Feng *et al*., 2023; Losso *et al*., 2025). These comparisons showed high agreement in some cases and low agreement in others. In this study, the hypocotyl vulnerability obtained with OV was consistently shifted towards higher Ψ than that obtained with AE for all species under study (Fig. 1; Supplementary Table S2). While this difference was minor for *A. pseudoplatanus* and *P. cembra*, it was higher for *L. decidua* (ΔΨ_OV50−AEmax_ 0.83 MPa) and particularly pronounced for *S. aucuparia* (ΔΨ_OV50−AEmax_ 2.50 MPa). A similar discrepancy between the two techniques has also recently been observed on several-years-old branches of three conifers, including *P. cembra* (Losso *et al*., 2025). The authors found that light was only transmitted at a maximum xylem depth of 150–200 μm, which is, however, more than the xylem thickness measured in all four seedlings under study (Table 2; Supplementary Fig. S3). One reason for the observed difference in the present study might be that less gel was applied to the exposed xylem for OV to ensure visibility of the xylem tissue, whereas a thicker gel layer was used for AE. Hence, the xylem visualized for OV was likely to be more exposed to air and, given the small size of the seedling hypocotyls, removal of the periderm exposed 30–50% of the hypocotyl xylem to air, potentially increasing the risk of air seeding (Sperry and Saliendra, 1994; Mayr *et al*., 2006; Venturas *et al*., 2019). This could have led to localized faster dehydration, reducing Ψ close to the sensor and causing the xylem to embolize earlier than if it had been protected by the periderm or by a thicker gel layer, as in the AE samples. Furthermore, although preliminary tests have shown uniform dehydration in seedlings of the same size (see ‘Materials and methods’), slight variations in Ψ may still occur between the undamaged proxy seedlings and the clamped ones with exposed xylem, which could lead to an underestimation of Ψ_OV50_. This may therefore explain the general shift of Ψ_OV50_ towards higher values compared with Ψ_AEmax_ for all species. In the specific case of *S. aucuparia*, which showed the most pronounced ΔΨ_OV50−AEmax_ (Fig. 1), the hypocotyl had a higher frequency in the small conduit diametric classes, especially those below 1 μm (Fig. 4). These may be difficult to observe with OV (Supplementary Fig. S2, where the smallest detected, and thus visible, embolism events were larger than 1 μm) and/or could be overshadowed by larger conduits (15–45 μm), but could still be detected with AE. Therefore, the failure to detect embolism in small conduits (which accounted for up to 14% of the total number of conduits) using OV may have led to an overestimation of the overall hypocotyl vulnerability of *S. aucuparia*, especially if small conduits are more resistant than larger ones (pit area hypothesis; Wheeler *et al*., 2005). In *L. decidua*, the overall smaller size of the conduits (Table 2) may also limit embolism detection with OV. However, the lack of overshadowing from larger conduits resulted in a less pronounced difference in ΔΨ_OV50−AEmax_ (see Fig. 1). Furthermore, while OV analysis is limited to the camera's field of view, AE has a greater spatial sensitivity that can detect acoustic signals several centimeters away from the acoustic sensor, explaining the observed differences (see also Losso *et al*., 2025). On the other hand, vulnerability analysis with AE does have its shortcomings as well. In particular, while defining the endpoint of dehydration is reliable for conifers, it remains critical for angiosperms, where acoustic activity may continue because other sources of signal (e.g. micro-fracturing of wood; Nolf *et al*., 2015; Rosner, 2015) are detected (see also ‘Materials and methods’). For future AE analysis, De Boeck and Steppe (2025) recently developed an objective method that can distinguish embolism-related signals more clearly from those of other sources, and help identify the endpoint of the vulnerability curve (i.e. 100% embolism). This method draws inspiration from previous work dealing with the same issues (Nolf *et al*., 2015; De Baerdemaeker *et al*., 2019; Oletic *et al*., 2020; Lamacque *et al*., 2022) and is based on an algorithm that clusters AEs (Sause *et al*., 2012; Vergeynst *et al*., 2016), as well as on the principle also used in the present study that Ψ_AEmax_ corresponds to 50% of loss of hydraulic conductivity (Ψ_50_). However, in three of the four species under study (*P. cembra*, *S. aucuparia*, and *L. decidua*), Ψ_AEmax_ was remarkably similar to Ψ corresponding to Ψ_50_ of older trees (see also below), as reported in previous studies using other techniques (Tissier *et al*., 2004; Lens *et al*., 2011; Charra-Vaskou *et al*., 2012; Choat *et al*., 2012; Losso *et al*., 2016, 2019; Feng *et al*., 2021). AE may therefore better reflect the vulnerability of the whole hypocotyl and all conduits, whereas OV focuses on the (hydraulically most important) outer xylem layers and larger conduits. For future studies, it would be interesting to use other advanced techniques, such as high-resolution micro-computed tomography imaging, to validate outcomes of AE and OV for seedlings of different species. For these reasons, and given that previous studies (Nolf *et al*., 2015; Lamacque *et al*., 2022; Beikircher *et al*., 2025) have also found that Ψ_AEmax_ provides results similar to those of hydraulic methods, we will use the acoustically obtained Ψ_AEmax_ to discuss the differences in vulnerability between the hypocotyl and the leaf in the following section.

### Hypocotyl and leaf hydraulic vulnerability

Vulnerability to drought measured by AE showed the lowest hydraulic thresholds in *A. pseudoplatanus* (Ψ_AEmax_ −2.01±0.07 MPa), while *S. aucuparia* (−3.95±0.16 MPa) and *P. cembra* (−3.83±0.10 MPa) were the most resistant to drought-induced xylem embolism (Fig. 1; Supplementary Table S2). The acoustically obtained Ψ_AEmax_ of *P. cembra*, *S. aucuparia*, and *L. decidua* was remarkably similar to the Ψ_50_ reported for branches collected from mature trees and measured with standard hydraulic techniques (Charra-Vaskou *et al*., 2012; Choat *et al*., 2012; Losso *et al*., 2016; Feng *et al*., 2021) (Supplementary Table S3). Beikircher *et al*. (2025) also reported a similar agreement between seedling hypocotyls and mature branches for a further four temperate tree species. However, the authors also found that the development of new xylem can alter the overall embolism resistance within a few weeks and thus vary considerably depending on the developmental stage of the seedling. This similarity may indicate an initial investment in safe xylem already at an early stage that is subsequently maintained also at older stages. Such an investment is crucial because at this early stage tree seedlings have low water storage capacity and small root systems, making them extremely dependent on water from the upper layers of the soil, which can easily dry out. *Pinus cembra*, *L. decidua* and *S. aucuparia* have a wide elevation range, and are among the few tree species reaching the Central Alps treeline. In particular, *P. cembra* can reach high elevations (up to 2390 m a.s.l.; Mattes, 1982) and is known to withstand harsh conditions (e.g. Mayr *et al*., 2003). Hence, seedlings of a few weeks old with low hydraulic vulnerability may be crucial for species recruitment and establishment in otherwise unfavorable environments (e.g. strong winds, high radiations, shallow soils). The low hypocotyl vulnerability of *P. cembra* was also reflected by the anatomy: (*t*/*b*)^2^ was almost twice as high (0.12±0.01, Table 2) as in the other species, and remarkably similar to that of branches collected from mature trees (Losso *et al*., 2016). This fits with the observed similarity in hydraulic vulnerability (see above) as in conifers (*t*/*b*)^2^ tends to correlate very well with increased resistance to drought-induced xylem embolism of both mature trees (Hacke *et al*., 2001) and seedlings (Beikircher *et al*., 2025). In contrast, the correlation between hydraulic vulnerability and (*t*/*b*)^2^ is less pronounced in angiosperm seedlings, and other anatomical traits such as pits, vessel length, and conduit connectivity (e.g. Lens *et al*., 2011; Li *et al*., 2016; Mrad *et al*., 2021; Pritzkow *et al*., 2022) may play a role. Accordingly, we observed less investment in cell wall reinforcement and hence comparatively thinner cell walls in *A. pseudoplatanus* and *S. aucuparia*, which may thicken at later stages of development. It should also be noted that at least some of the conduits analysed were still under development (Supplementary Fig. S3) and that the protoxylem can differ considerably from the metaxylem and the secondary xylem, for example in the thickening pattern of the lignified secondary cell wall (Růžička *et al*., 2015).

Of the four species under study, *A. pseudoplatanus* is the most studied, but the vulnerability data in the literature are quite heterogeneous, making comparisons between seedlings and older trees somewhat difficult. While some studies using standard hydraulic techniques on branches reported Ψ_50_ (−1.6 MPa in Tissier *et al*., 2004; −2.2±0.2 MPa in Lens *et al*., 2011) similar to the acoustically obtained Ψ_AEmax_ (−1.86±0.12 MPa; Fig. 1), others on 6-month-old (−2.81 MPa; Losso *et al*., 2019) and 2-year-old plants (−3.70 MPa; Lübbe *et al*., 2017) measured more negative Ψ_50_. However, Ψ_AEmax_ fell between Ψ_AEmax_ reported for 4-week-old (−1.27±0.09 MPa) and 8-week-old (−1.87±0.04 MPa) seedlings in a recent study (Beikircher *et al*., 2025). This combination of data further emphasizes that the hydraulic vulnerability of seedlings depends on xylem development and that it can change significantly within a matter of weeks. *Acer pseudoplatanus* seedlings seem to decrease their Ψ_50_ during the first few years of growth, or even within a few months (see Losso *et al*., 2019; Beikircher *et al*., 2025), and then reduce this investment as the root system extends into deeper soil layers and the hydraulic capacitance increases. This shift in hydraulic vulnerability during seedling development may also be indicated by the acoustic activity of all four species showing additional peaks at higher Ψ (Fig. 1), which may be related to embolism formation in more vulnerable primary xylem conduits (Choat *et al*., 2005, 2015; Venturas *et al*., 2019; Miller *et al*., 2020; Haverroth *et al*., 2024), as previously suggested by Beikircher *et al*. (2025).

In seedlings, in addition to safe hypocotyl hydraulics, maintaining leaf performance is also critical for their survival, as the photosynthetic activity relies on only cotyledons and/or a few true leaves. Leaf vein Ψ_OV50_ measured with optical technique revealed a significantly lower vulnerability in *A. pseudoplatanus* (−1.95 MPa) than in *S. aucuparia* (−0.81 MPa; Fig. 3; Table 1). *Acer pseudoplatanus* leaf vein Ψ_OV50_ was also lower than that of the hypocotyl, whereas the opposite was observed for *S. aucuparia* leaf veins and hypocotyl (Table 1). For seedlings, having safe leaves at this early stage is extremely beneficial as they are the first true leaves and will be important in supporting initial growth. However, measurements on *S. aucuparia* were done on the terminal leaflet of the compound leaves, which were probably still developing and had a higher hydraulic vulnerability. As previously reported in a study of the compound leaves of six herbaceous and one tree species (Rimer and McAdam, 2024), it should be noted that differences in hydraulic vulnerability within the leaflets of the same leaf are expected to be negligible. However, further investigation of newly developing leaves of seedlings may still be worthwhile. Interestingly, for both species, the percentage of embolized area (i.e. of embolized xylem) increased steeply within a small range in Ψ (Fig. 3). In *A. pseudoplatanus*, the percentage of embolized area changed from values close to 0% to values close to 100% in even less than 0.5 MPa. Such a rapid increase in embolized xylem indicates a fast increase in embolism propagation when critical Ψ is reached (e.g. Domec and Gartner, 2001). Hence, seedlings may be safe up to this narrow threshold, but then suddenly be at high risk of embolism, whereas a wider vulnerability range (as often found in mature trees) may allow some residual transport capacity even if parts of the xylem are embolized. Further research on the hydraulic vulnerability of developing seedling leaves is needed to shed light on the hydraulic architecture and coordination between organs of developing tree seedlings.

## Conclusion

The observed hypocotyl vulnerability of the study seedlings, which was remarkably similar to that of mature tree branches for three out of four species, and their coordinated hydraulic architecture, clearly demonstrate the importance of an early investment in hydraulic structures and functions to prevent xylem embolism. This investment is all the more important as small seedlings are unlikely to recover or refill if they are critically embolized. In the context of climate change, where longer and more frequent droughts and higher temperatures (and thus increased vapor pressure deficit and transpiration) are expected, it will be crucial for (natural) seedling establishment to have sufficiently high drought resistance. However, further research on other species and phenological stages, as well as on the hydraulic architecture at different stages of development and the coordination with other drought-related traits (e.g. rooting system, stomatal regulation) deserves attention, as it will be important for our understanding of both forest regeneration and restoration.

## Supplementary Material

erag039_Supplementary_Data

## Data Availability

The primary data supporting this study were not made publicly available at the time of publication. The data that support the findings of this study are available from the corresponding author upon request.

## Supplementary data

The following supplementary data are available at *JXB* online.

Fig. S1. Example photos showing the experimental setup with the acoustic sensors and OV clamps installed on *A. pseudoplatanus* seedlings.

Fig. S2. Visual representation of the embolized xylem of hypocotyls of *Acer pseudoplatanus*, *Sorbus aucuparia*, *Pinus cembra*, and *Larix decidua* recorded during dehydration by OV at different water potentials (MPa).

Fig. S3. Hypocotyl cross and longitudinal sections of *Acer pseudoplatanus*, *Sorbus aucuparia*, *Pinus cembra*, and *Larix decidua*.

Table S1. Age, height and basal hypocotyl diameter of *A. pseudoplatanus*, *S. aucuparia*, *P. cembra*, and *L. decidua* seedlings.

Table S2. Water potential at maximum acoustic activity (Ψ_AEmax_) and at 50% of embolized pixel area (Ψ_OV50_), and the difference between the two (ΔΨ_OV50−AEmax_) of hypocotyls of *Acer pseudoplatanus*, *Sorbus aucuparia*, *Pinus cembra*, and *Larix decidua* seedlings.

Table S3. Hypocotyl water potential at maximum acoustic activity (Ψ_AEmax_) measured in this study and water potential of mature branches at 50% loss of conductivity (Ψ_50_) reported by older studies, with corresponding reference, of *A. pseudoplatanus*, *Sorbus aucuparia*, *Pinus cembra*, and *Larix decidua*.
