# Supplementary material for: Unraveling the hydraulic vulnerability of tree seedlings using optical and acoustic techniques
Source: J Exp Bot. 2026 Jan 29;77(10):2970–81. doi: 10.1093/jxb/erag039 (PMC13187666; doi:10.1093/jxb/erag039)

Supplemental information

Supplementary Table S1. Age, height and basal hypocotyl diameter of *A. pseudoplatanus*, *S. aucuparia*, *P. cembra* and *L. decidua* seedlings. Mean ± SE.

	Age, week	Height, cm	Diameter, mm
Acer pseudoplatanus	4.8	6.24 ± 0.15	1.75 ± 0.07
Sorbus aucuparia	7.1	6.70 ± 0.24	1.50 ± 0.10
Pinus cembra	5.8	3.70 ± 0.13	1.72 ± 0.07
Larix decidua	7.6	4.60 ± 0.17	0.81 ± 0.03

Supplementary Table S2. Water potential at maximum acoustic activity (Ψ_{AEmax}) and at 50% of embolized pixel area (Ψ_{OV50}), and the difference between the two ($\Delta\Psi_{OV50-AEmax}$) of hypocotyls of *Acer pseudoplatanus*, *Sorbus aucuparia*, *Pinus cembra* and *Larix decidua* seedlings. n indicates the number of replicates. Mean ± SE.

	**Technique**	**Ψ ± SE, MPa**	**$\Delta\Psi_{OV50-AEmax}$, MPa**	**n**
Acer pseudoplatanus	Ψ_{AEmax}	-1.86 ± 0.12 a	0.31	8
	Ψ_{OV50}	-1.55 ± 0.06		6
Sorbus aucuparia	Ψ_{AEmax}	-3.95 ± 0.16 b*	2.50	8
	Ψ_{OV50}	-1.46 ± 0.09		7
Pinus cembra	Ψ_{AEmax}	-3.83 ± 0.10 b	0.48	8
	Ψ_{OV50}	-3.35 ± 0.11		8
Larix decidua	Ψ_{AEmax}	-2.84 ± 0.21 c*	0.83	6
	Ψ_{OV50}	-2.01 ± 0.07		7

Different letters indicate statistically significant differences between Ψ_{AEmax} of different species. Asterisks indicate significant differences between Ψ_{OV50} and Ψ_{AEmax} of the same species.

Supplementary Table S3. Hypocotyl water potential at maximum acoustic activity (Ψ_{AEmax}; ± SE) measured in this study and mature branches water potential at 50% loss of conductivity (Ψ_{50}) reported by older studies, and corresponding reference, of *A. pseudoplatanus*, *Sorbus aucuparia*, *Pinus cembra*, and *Larix decidua*. Mean ± SE or ranges.

**Species**	**Ψ_{AEmax}, MPa**	**Ψ_{50}, Mpa**	**Reference**
A. pseudoplatanus	-1.86 ± 0.12	-1.6	Tissier *et al*., 2004
		-2.20 ± 0.21	Lens *et al*., 2001
S. aucuparia	-3.95 ± 0.16	-3.40 to -5.12	Charra-Vaskou *et al*., 2012
		-3.19 to -4.19	Choat *et al*., 2012
P. cembra	-3.83 ± 0.10	-2.38 to -2.95	Losso *et al*., 2016
		-3.57 to -3.58	Feng *et al*., 2021
		-3.02 to -4.18	Choat *et al*., 2012
L. decidua	-2.84 ± 0.21	-3.66 to 3.75	Feng *et al*., 2021
		-2.85 to -3.61	Charra-Vaskou *et al*., 2012
		-3.60 to -4.45	Choat *et al*., 2012

Supplementary Fig. S1. Example photos showing the experimental setup with the acoustic sensors (A) and OV clamps (B) installed on *A. pseudoplatanus* seedlings. Additional seedlings were placed in between the other samples (A-B) for Ψ measurements. In panel C, clamps were positioned on a leaf and stem on the same seedling for simultaneous OV measurements. All seedlings were covered with black plastic bags to maintain a balanced Ψ within plants.

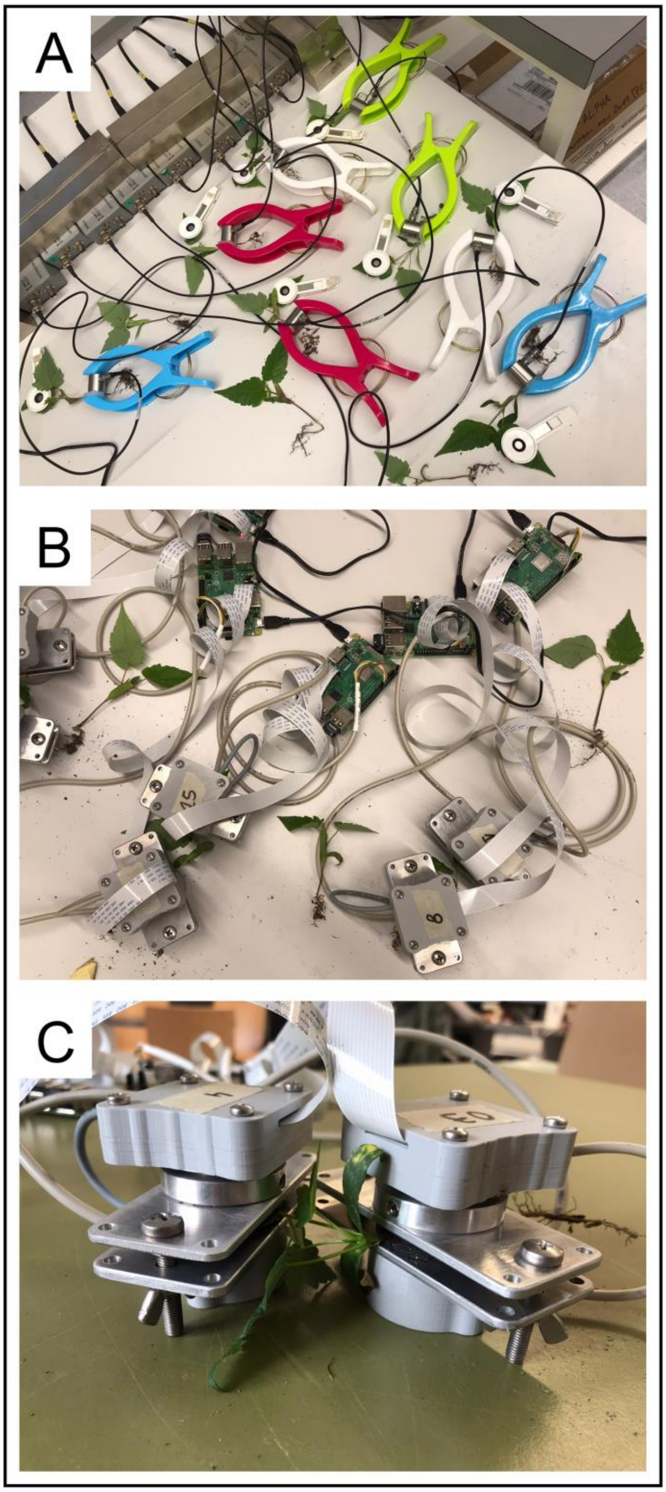

Supplementary Fig. S2. Visual representation of the embolized xylem of hypocotyls of *Acer pseudoplatanus*, *Sorbus aucuparia*, *Pinus cembra* and *Larix decidua* recorded during dehydration by OV at different water potentials (MPa). White bars in each panel indicate a 3 mm reference scale.

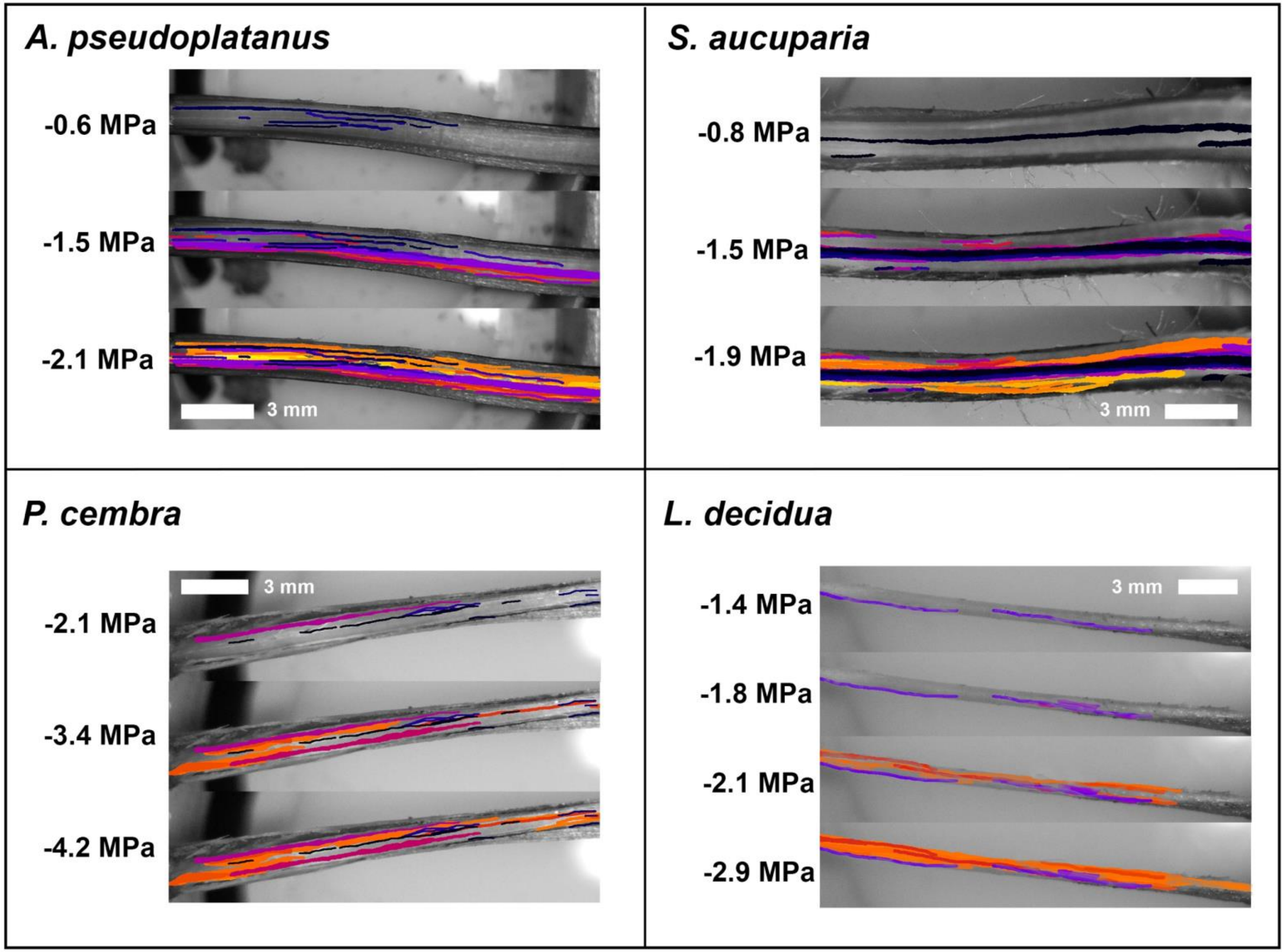

Supplementary Fig. S3. Hypocotyl cross and longitudinal sections of *Acer pseudoplatanus*, *Sorbus aucuparia*, *Pinus cembra* and *Larix decidua*. Sections were stained with safranin and astra blue, with red staining indicating mainly lignified structures (xylem, sclerenchyma). For longitudinal sections, the orientation of the samples is bark on the left and pith on the right. Black bars in each panel indicate a 50 µm reference scale.

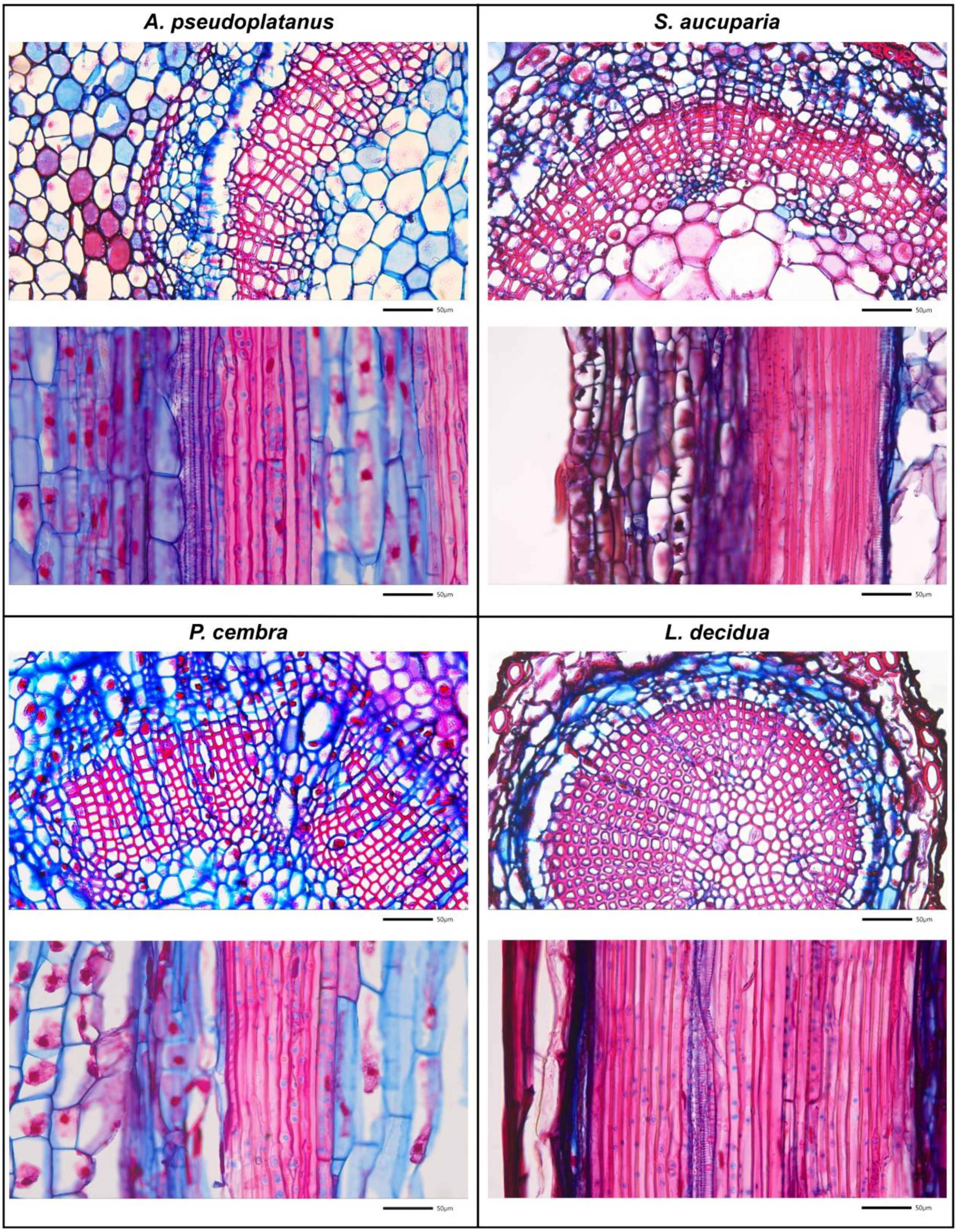

Supplement: erag039_Supplementary_Data [file erag039_supplementary_data.pdf]
